# Bioelectrical Impedance Vector Analysis of Young Elite Team Handball Players

**DOI:** 10.3390/ijerph182412972

**Published:** 2021-12-08

**Authors:** Andrea Di Credico, Giulia Gaggi, Anastasios Vamvakis, Sofia Serafini, Barbara Ghinassi, Angela Di Baldassarre, Pascal Izzicupo

**Affiliations:** 1 Department of Medicine and Aging Sciences, University “G. D’Annunzio” of Chieti-Pescara, 66100 Chieti, Italy; andrea.dicredico@unich.it (A.D.C.); giulia.gaggi@unich.it (G.G.); sofiaserafini97@gmail.com (S.S.); b.ghinassi@unich.it (B.G.); izzicupo@unich.it (P.I.); 2 Beth Israel Deaconess Medical Center, Harvard Medical School Initiative for RNA Medicine, Harvard Medical School, Boston, MA 02115, USA; 3 3rd Department of Pediatrics, Medical School, Aristotle University of Thessaloniki, Hippokration General Hospital, 54649 Thessaloniki, Greece; tvamvakis@yahoo.gr

**Keywords:** team handball, phase angle, BIVA, body composition, youth sport, tolerance ellipses

## Abstract

Team handball is a highly dynamic sport where physical demands differ between categories and roles. Thus, physical characteristics are fundamental for the final performance. This study aims to (a) characterize a sample of young male and female elite team handball players with a non-athletic reference population; (b) to generate their 50%, 75%, and 95% percentiles of the bioelectrical variables. The study included 55 young elite team handball players (Males, *n* = 37, age = 17.0 ± 1.2 yrs, height = 185.8 ± 7.3 cm, weight = 82.0 ± 11.0 kg, body mass index (BMI) = 23.7 ± 2.5; Females, *n* = 18, age = 17.8 ± 0.9 yrs, height = 171.2 ± 6.4 cm, weight = 67.4 ± 7.2 kg, BMI = 23.0 ± 2.0). Height and bioelectrical variables were assessed in a state of euhydration and standard conditions. Bioelectrical impedance vector analysis (BIVA) was used to characterize the bioelectrical vector (BIA vector) distribution pattern for each group. Compared to the reference values, BIA vector showed statistically significant differences in males U17 (*n* = 19, T^2^ = 51.0, *p* < 0.0001), males U19 (*n* = 18, T^2^ = 82.0, *p* < 0.0001) and females U19 (*n* = 18, T^2^ = 85.8, *p* < 0.0001). Male groups were also bioelectrically different (T^2^ = 13.7, *p* = 0.0036). BIVA showed specific bioelectrical characteristics in young male and female elite handball players. This study provides an original data set of bioelectrical impedance reference values of young male and female elite team handball players. Our result might help to interpret individual bioimpedance vectors and define target regions for young handball players.

## 1. Introduction

Team handball is a dynamic sport game played at professional and youth levels worldwide. Previous studies indicated that handball is characterized by high-intensity movements and strategic and technical actions, such as sprints, stops, changes of direction, throws on goal, passes, jumps and body tackles, interspersed with actions necessary for recovery, like walking and standing [1,2]. This implies that handball highly taxes both the cardiovascular and neuromuscular systems, stimulating favorable adaptations regarding oxygen utilization and skeletal muscle capacities [3,4,5]. The relative workload of team handball is about 70–80% of the maximal oxygen uptake (VO_2_max), and the total distance covered per full-time match (60 min) ranges between 3900 to 4700 m, of which about 700 to 1500 m are related to the strategic/technical changes mentioned above [1,2,6]. Physical demands differ between elite and lower-level players, and also depend on playing court [7,8,9] and playing positions, with wing players performing faster breaks, and backcourt players and pivots experiencing more physical impacts with opponent players [1,2]. Thus, physical characteristics that are dictated both by genetics and training stimuli [10,11,12,13] influence playing performance since they are highly related to the playing positions [2,14] and competitive level in both males [15] and females [16].

The few studies describing physiological and anthropometric profiles of young elite handball players suggest that the skills and fitness components required to perform at high level are essentially the same needed for adults [17,18,19]. For example, Ingebrigtsen et al. showed that both male and female under sixteen and under eighteen years old (U16 and U18) players were taller but lighter than nonathletic age-matched adolescents, and lighter than senior players. Furthermore, they found very few differences in performance among U16, U18, and senior teams [18]. On the other hand, Ruscello et al. found that Italian junior national team players showed worse anthropometrics and performance attributes than seniors [14]. Overall, these studies suggest that the higher the national ranking (e.g., Norwegian vs. Italian national teams), the closer the physical and performance profiles between juniors and seniors are. For this reason, physical and performance profiles become relevant at the early stages of talent identification and selection as well as for physical development programs at more advanced stages. Bearing in mind this evidence, the monitoring of body composition, nutritional and hydration status, and growth at the youth level became fundamental practices.

Bioimpedance analysis (BIA) is a fast and noninvasive technique used in healthcare and sports. In BIA, the impedance, or the opposition to the flow of an electric current through body tissues, is measured through the injection of an alternating electrical current pulse of harmless intensity into the body. In conventional BIA, impedance and its components (i.e., resistance [R] and reactance [Xc]) are applied to simple or multiple regression equations for body compartments (e.g., fat mass [FM] and fat-free mass [FFM]) estimation [20,21]. However, using prediction equations can present some limitations, such as assuming a fixed hydration coefficient for FFM [20,21], especially in assessing younger subjects [22]. These and other limitations can be overcome through the bioelectrical impedance vector analysis (BIVA) approach proposed by Piccoli et al. [23]. BIVA interpolates R and Xc, standardized for the subject height, on the RXc graph as a single vector, to assess soft tissues and body fluids. This qualitative analysis allows comparison of the position of the vector to the tolerance ellipses (representing the population reference values) and to study its displacements by collecting multiple measurements (e.g., during a season [24], after exercising [25], or over an intervention program [26]). Vector elongation or shortening over time represent decreases or increases in hydration, while lateral displacement to the left or the right reflect gains or losses in the soft tissues, respectively [23].

Another important parameter is the phase angle (PA), still derived from resistance and reactance as the arctangent of Xc/R × 180°/π. It describes the lag between voltage and current and is directly measured by phase-sensitive devices, using the geometric relationship between R and Xc [27]. PA has been interpreted as an index of membrane integrity and water distribution between the intracellular and extracellular compartments, recently also in athletes [28,29].

Athletes typically possess a higher soft tissue mass and different fluid content than the general population [28,29,30], even at the youth level [25,28,31]. However, although bioelectrical impedance standards are established for the normal healthy youth population or in clinical settings, they are not available for team handball and many other youth sports. Thus, filling this gap can help both in selecting and developing talents. Furthermore, this may be even more relevant in handball, since the anthropometric values of young players are early close on to those of adults [18]. On these premises, this study aimed (a) to characterize a sample of young male and female elite team handball players in relation with a nonathletic reference population; and (b) to generate their 50%, 75%, and 95% percentiles of the bioelectrical variables. We hypothesized that a specific distribution of BIVA variables would characterize young handball players compared to the reference population. A further aim of the present study was to compare the anthropometric profile of the players according to their positional roles. We hypothesized that wingers were shorter and lighter than other players.

## 2. Materials and Methods

### 2.1. Subjects

This study included 55 young male and female elite team handball players, selected for simultaneous training camps of the male U17 (*n* = 19) and U19 (*n* = 18), and female U19 (*n* = 18) Italian Handball National Teams. Inclusion criteria were as follows: (1) to have competed at national and/or international level in the previous year; (2) absence of injuries or any clinical condition at the time of the study; (3) for females, to be in a postmenarcheal state; (4) to not be under contraceptive or menstrual cycle pharmacological regulator treatment. The technical staff of the national teams approved the conduct of the study, with parental permission when needed. Thus, the present data arose as a condition of the monitoring procedures regularly performed by the respective national teams at the beginning of the training camps. Because of the retrospective nature of the analyses without interfering in the training routine, signatures of the informed consent form were not required [32]. The procedures were performed in accordance with the ethical standards of the Helsinki Declaration. Nevertheless, all physical performance data were anonymized before analyses to ensure player confidentiality.

### 2.2. Design

The study was conducted two weeks before the youth European Handball Championships. At the time of evaluation, participants were within the 4-week precompetitive mesocycle. Demographic information (years of practice and positional role) was collected upon the arrival of the athletes. Players were required to abstain from caffeine, alcohol, and exercise the day before BIA measurements to attain a state of euhydration. They were also instructed to drink 3.0 L of fluid over 24 h (2.0 L to be consumed between 6:00 p.m. and 10:00 p.m.) in addition to their habitual dietary practices. From 10:00 p.m. until the start of the assessment on the following day, no other fluid or food intake was allowed [25,30,33,34]. On the day of the assessment, from 7:00 a. m. to 8:00 a.m., pretraining anthropometric and bioelectrical data (R, R/H, Xc, Xc/H, PA, Z, and ECW: TBW) measurements were performed in a thermoneutral room (25 °C), after voiding, in light clothing, and without conducting garments.

### 2.3. Anthropometry and Bioelectrical Impedance Analysis

Height (H) was recorded to the nearest 0.1 cm with a stadiometer (Seca 220, Hamburg, Germany) and weight was measured to the nearest 0.1 kg with a high-precision mechanical scale (Seca 710, Hamburg, Germany), as previously described [35]. Body mass index (BMI) was calculated as body mass/height^2^ (kg/m^2^). Anthropometric measurements were carried out according to the standard criteria of The International Society for the Advancement of Kinanthropometry [36]. The impedance measurements were performed using BIA (BIA 101 Anniversary, Akern, Florence, Italy) with an electric current at a frequency of 50 kHz. The device was calibrated in the morning following the manufacturer’s instructions. Measurements were made on an isolated cot from electrical conductors. Participants were lying in the supine position with a leg opening of 45° compared to the median line of the body and the upper limbs, distant 30° from the trunk. After cleaning the skin with alcohol, two electrodes (Biatrodes Akern Srl, Florence, Italy) were placed on the right hand back and two electrodes on the neck of the corresponding foot [23]. Bioimpedance vector analysis was carried out using the BIVA method, normalizing resistance (R) and reactance (Xc) parameters for H, in meters [23]. Bioelectrical phase angle (PA) was calculated as the arctangent of Xc/R × 180°/π [27].

### 2.4. Statistical Analysis

Descriptive statistics (mean, SD) were calculated for each independent variable and age and sex category, while the normality of the data was verified applying the Shapiro–Wilk test. Normally distributed data were compared by one-way analysis of variance (ANOVA). When a significant F ratio was obtained, Bonferroni post hoc test was used to evaluate the differences among the groups. Since the R/H, body cellular mass (BCM), and ECW: TBW did not show a normal distribution, differences among the three groups were analyzed using the Kruskal–Wallis test. In this case, post hoc testing was performed using the Mann–Whitney U test. Pearson’s correlation coefficient was used to determine possible statistical associations among variables. Spearman’s rank correlation coefficient was applied in the case of non-normally distributed variables. Each player was plotted in the tolerance ellipses (50%, 75%, and 95%) of the 14- to 15-year-old healthy male and female Italian reference population. Compared to our sample, these populations represent the closest references in terms of age [37]. A two-sample Hotelling’s T^2^ test was used to determine the BIA vector differences between U17 and U19 male players and differences for each group vs. the reference populations [37]. Furthermore, we examined the differences vs. basketball [28] and soccer [31] players of similar age and level. Distances between ellipses were calculated by the Mahalanobis test [38]. The Mahalanobis distance (D) is a descriptive statistic that provides a relative measure of data-point distances (residual) between vectors. Separate 95% confidence ellipses indicate a significant vector difference. A *p*-value < 0.05 was considered significant. IBM SPSS 23.0 (SPSS, Chicago, IL, USA) and BIVA software [38] were used for all statistical calculations. In particular, BIVA software allows to plot individuals in the tolerance ellipses (50%, 75%, and 95%) of a reference population. These ellipses are obtained from the literature using the population size, mean, and SD of both R/H and Xc/H, with their linear correlation coefficient. Furthermore, BIVA software allows the calculation of the two-sample Hotelling’s T^2^ test and the Mahalanobis D, by means of the same descriptive variables.

## 3. Results

### 3.1. Sex, Age Category and Positional Roles Comparisons

Apart from BMI and FM, significant differences emerged for the investigated variables among the three groups. Table 1 shows the whole sample and subgroups values and the differences between them.

The comparison of positional roles in males (Table 2) indicates that wingers were shorter and lighter than goalkeepers, backs, and pivots. Furthermore, they had less robustness, as indicated by the FFM, BCM and the lowest values of adiposity. Centers showed lower values than pivots in terms of height, body weight, and adiposity. Regarding the PA, centers showed higher values than goalkeepers and pivots. Backs and wingers showed higher PA values than goalkeepers. Due to the small size of positional role groups in females, no statistical comparison was performed.

### 3.2. Bioelectrical Impedance Vector Analysis and Comparisons

**Figure 1:**
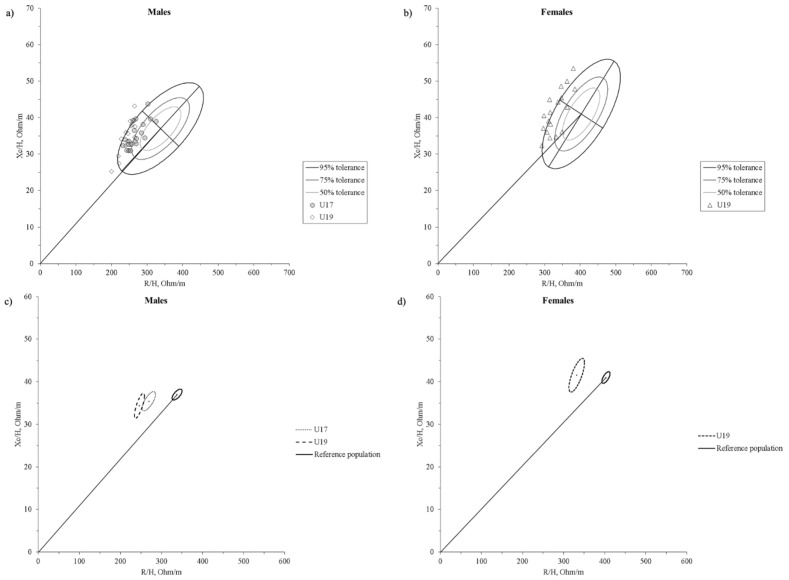
Scattergrams of the (**a**) male (U17 and U19) and (**b**) female U19 Italian youth team handball selections. Individual impedance vectors plotted on the 50%, 75%, and 95% tolerance ellipses of the corresponding healthy male and female reference populations [37]. The 95% confidence ellipses for the mean impedance vectors of the (**c**) male (U17 and U19) and (**d**) female U19 Italian youth team handball selections and the healthy male and female reference populations [37] are shown. R/H, height-adjusted resistance; Xc/H, height-adjusted reactance.

The BIVA point graph (Figure 1a,b) indicates that young handball players mainly fell outside the 75% tolerance ellipse regardless of age or sex; they were outside the 95% tolerance ellipse in many cases.

**Figure 2:**
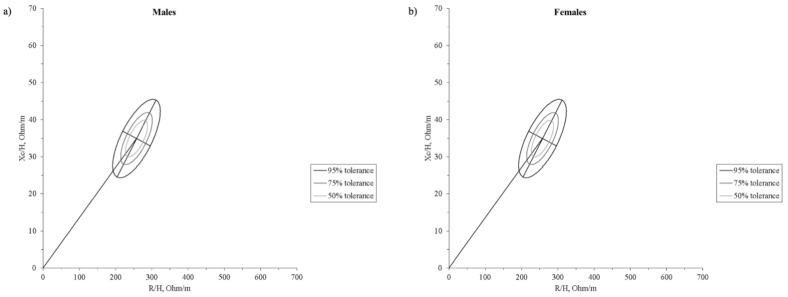
Tolerance ellipses at 50%, 75%, and 95% generated for (**a**) the male (U17 and U19) and (**b**) female (U19) youth team handball squads. R/H, height-adjusted resistance; Xc/H, height-adjusted reactance.

Compared to the reference values, BIA vector showed statistically significant differences in males U17 (T^2^ = 51.0, D = 1.79, *p* < 0.0001), males U19 (T^2^ = 82.0, D = 2.32, *p* < 0.0001) (Figure 1c) and females U19 (T^2^ = 85.8, D = 2.34, *p* < 0.0001) (Figure 1d). Male groups were also bioelectrically different (T^2^ = 13.7, D = 1.22, *p* = 0.0036) (Figure 1c). Figure 2 shows the 50%, 75%, and 95% tolerance ellipses corresponding to the males’ (*n* = 37, R/H = 258.0 ± 25.0 Ω/m; Xc/H = 34.9 ± 4.0 Ω/m; r = 0.69) and females’ (*n* = 18, R/H = 331.9 ± 28.8 Ω/m; Xc/H = 41.5 ± 6.0 Ω/m; r = 0.73) samples.

**Figure 3:**
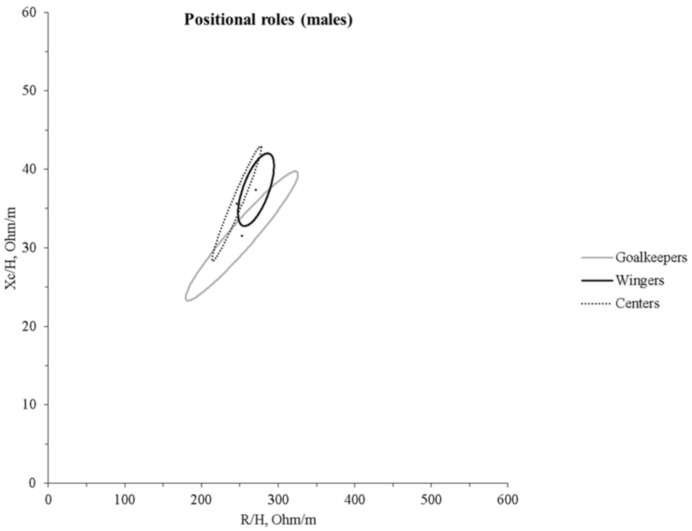
The 95% confidence ellipses for the mean impedance vectors of goalkeepers, winger, and centers. R/H, height-adjusted resistance; Xc/H, height-adjusted reactance.

The BIA vector comparison based on the positional roles in males indicates that wingers and centers differ from goalkeepers (T^2^ = 8.3, D = 1.52, *p* = 0.048, T^2^ = 21.3, D = 2.66, *p* < 0.005, respectively), with wingers showing a longer vector, goalkeepers the shorter, while the centers’ vector was more to the left (Figure 3). Due to the small size of positional roles groups in females, no comparison was performed.

**Figure 4:**
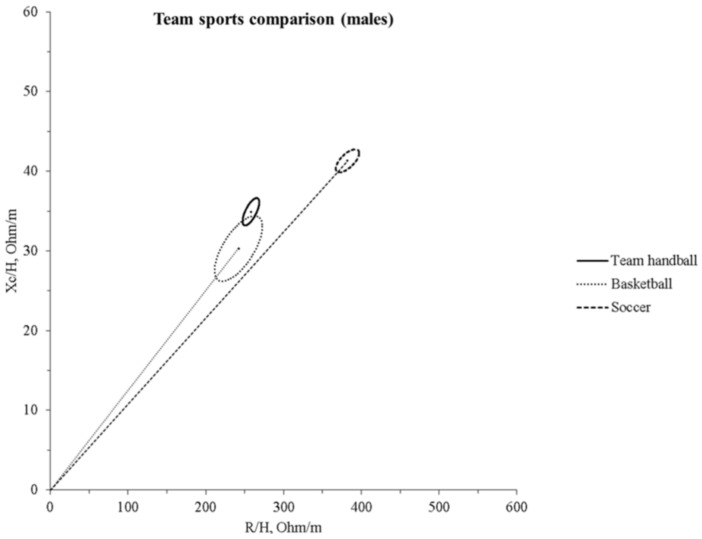
The 95% confidence ellipses for the mean impedance vectors of the male Italian youth team handball selections and the basketball [28] and soccer [31] reference populations are shown. R/H, height-adjusted resistance; Xc/H, height-adjusted reactance.

Compared to other athletic populations, our players significantly differ from both basketball [28] (T^2^ = 11.2, D = 0.93, *p* = 0.006) and soccer [31] (T^2^ = 86.6, D = 1.68, *p* < 0.0001) players (Figure 4).

### 3.3. BIVA Correlations

ECW: TBW correlates negatively with age and PA in males (*n* = 37, r_s_ = −0.486, *p* = 0.002; r_s_ = −0.844, *p* < 0.001, respectively) and PA in females (*n* = 18, r_s_ = −0.994, *p* < 0.001).

## 4. Discussion

This study indicates that young male and female elite team handball players represent a specific population, with bioelectrical impedance values that differ from the general age-matched populations. No differences emerged on anthropometric parameters between male U19 and U17 players, while female players were lighter and shorter and with higher Xc/H values than males. Although BMI was similar between the three groups, females showed lower FFMI and higher FMI. Furthermore, U19 male players showed higher PA and ECW:TBW than the other two groups, probably denoting a better cell condition and greater intracellular water content due to muscle hypertrophy [29]. In addition, we found specific BIA vector distributions in U19 and U17 male and U19 female players. Finally, male wingers and centers showed a smaller body size. However, the difference in FFM was not confirmed when adjusted for body height (FFMI). Centers showed the highest PA values, followed by wingers and backs, and they also revealed the BIA vector located on the left compared to the other positional roles. Thus, centers and wingers showed a better body composition (lower FM%) and soft tissues mass quality (higher PA, and lower ECW:TBW), despite the body size. These results can be obviously explained for wingers as they are the most dynamic players. For this reason, they are also leaner than other positional roles. On the other hand, further studies are needed to confirm the remaining differences found in this study.

Since our samples were one year older than the Norwegian counterparts [18] (U17 vs. U16 and U19 vs. U18), body height can be considered in line with previously published data. In contrast, body weight and BMI differences are more pronounced. Our sample was heavier, particularly concerning U19 male and female players, and the age mismatch between the samples of the two studies may only in part explain this difference. Previous studies reported FM% in adult handball players of different levels, with values ranging from ~11% to ~17% for males [17,39] and ~19% to ~28% for females [16], which places our sample in the upper limit of the reference values for handball players. In addition, we noticed important differences among positional roles, with the wingers in lower limit indicated from previous studies and goalkeepers and centers showing even higher values. However, considering that no FM values are indicated for the Norwegian sample in the study of Ingebrigtsen et al., and that different methods and equations were adopted among studies, we cannot conclude that our sample had higher fat mass than their counterparts in other studies.

In general, a gradient ranging from the female group to the U17 and U19 male counterparts can be observed, with the first showing the less robust physical structure and the last showing the more robust. While a significant difference between male and female was expected, investigating the difference between U17 and U19 teams was considered relevant, taking into account that youth players in the highest positions of the national team rankings show fewer differences from the older ones [18]. These differences can be attributed to the sex, as boys develop greater muscle masses and body structures than females due to distinctive sexual and hormonal maturation [40], and due to chronological age [41], as well as to higher levels of physical activity in males than females, independently from sports participation [42]. Indeed, the male U19 group presented higher FFM and FFMI, BCM, and PA values than the younger counterparts and females. On the other hand, the younger male players did not differ from the older female counterparts in PA and ECW:TBW values, probably because of the different growth rate trajectories between sexes [40]. Furthermore, differences in male groups may be indicative of physique-based selection and more prolonged training exposure. A confirmation in this direction is furnished by the increasing trend in FFM and BCM values towards U19 males, the inverse trend in the R/H component, and the correlations of ECW: TBW with age and PA found in our study. Indeed, recent studies indicated that bioelectrical impedance parameters (PA, ECW: TBW, ICW) might indicate bone and muscle growth, maturation, and performance capacity [31,43,44]. Consequently, the mean impedance vector of the U19 male group was significantly displaced to the left than that of the other two groups, while the mean impedance vector of the U19 female group was more to the right on the RXc graph. Monitoring bioelectrical impedance parameters may indicate athletes’ potential, as it has been recently shown that PA correlates with sprint performance in young soccer players [44]. Notably, positional roles in handball, as well as in other team sports, require different skills, with wingers generally being faster than other players and central offensive and defensive players (in team handball pivots and centers, respectively) stronger. Thus, the fact that the wingers and centers showed the highest PA values in our study is in line with previous research works that found specific anthropometric profiles in different positional roles based on the skills required [14,45]. In recent years, BIVA has been widely used in sport to evaluate acute changes in hydration [25] or track hydration and body composition during a season [24]. Furthermore, an increasing number of studies reported specific tolerance ellipses for the athletic populations [25,28,29,30,31,46]. Our sample differed from young soccer [31] and basketball [28] players presented in other studies, denoting the need for specific tolerance ellipses for handball players (Figure 4). The difference among handball, basketball, and soccer players can be explained by considering that body height is a selective and determining factor in basketball, while body structure and, to some extent, body height are essential in handball. In fact, our sample was shorter than the basketball players in the study by Koury et al. [28], and this difference might explain why the latter showed a shorter vector. In the BIVA method, R and Xc are standardized for body height and plotted on the RXc graph. Thus, this morphological characteristic can be a key element for the differences in specific tolerance ellipses across sport populations. Indeed, Campa et al. [30] showed that volleyball players (another sport in which height is selective and determinant) significantly differ from soccer players [29].

Considering the distribution of the data points of our sample on BIVA tolerance ellipses of the reference population [37], the majority of the athletes was outside the 75% tolerance ellipse and the 95% tolerance ellipse in many cases. In particular, the whole sample was shifted on the left side, and most of the players below the short axis of tolerance ellipses, probably due to the relatively high values of lean body mass, muscle glycogen reserves, and plasma volume commonly found in athletes [47]. Furthermore, Campa et al. [48] recently suggested that athletes presenting a higher endomorphic component have a lower vector. In contrast, those with a larger mesomorphic component display higher vector inclinations on the RXc graph. In team handball, goalkeepers tend to show the higher FM and endomorphic component, while centers tend to be lean, with the highest values of the mesomorphic component [45]. According to this observation, in our study, goalkeepers had the lower vector, while centers displayed higher inclination, resulting in the RXc graph’s left extreme.

Overall, this study has some strengths: first, it fills the gap in bioelectrical impedance standards for youth handball players. Therefore, the tolerance ellipses from our study can be adopted as a reference for evaluation and comparison of young handball players. Furthermore, after the identification of the weak points of the body structure, appropriate training interventions can be planned, targeting specific regions of the RXc graph derived from this study. Secondly, measurement conditions that potentially can affect hydration and bioelectrical impedance parameters [33,34] were strictly controlled. However, this study also has some limitations. First, the sample size is relatively small, particularly for female participants. However, it is worth noting that the sample represents a national selection of the most talented Italian players, and as such, it is very specific. Secondly, the current ranking of the Italian male and female youth handball teams is relatively low. Thus, we can speculate that higher-ranked teams may have a mean vector placed even more to the left on the RXc graph. However, to our knowledge, this is the first study to provide bioimpedance tolerance ellipses for young handball players and therefore it offers a benchmark for anyone wishing to approach BIVA in this sport at the youth level.

### Practical Applications

This study provides BIA data of an elite sample of young male and female handball players, which may help determine target regions of bioelectrical impedance vectors for young handball players. As a result, trainers can plan specific training programs to induce appropriate vector migration [21,24,25,26].

## 5. Conclusions

In conclusion, this study indicates that young handball players have specific tolerance ellipses, high FFM and BCM, and increasing robustness from female to U17 and U19 male players. In addition, BIVA ellipses suggested that sex, chronological age, intense training routine, and physique-based selection may determine the specificity of bioelectrical impedance properties of handball players.

## Figures and Tables

**Table 1:** Anthropometric, body composition, and bioelectrical data of the whole sample of young male and female elite team handball players and separated by sex and age category.

Variable	Males U17 *n* = 19	Males U19 *n* = 18	Females U19 *n* = 18	Whole Sample *n* = 55	F	Χ **^2^**	*p*-Value
Age (years)	16.5 ± 0.6	18.6 ± 0.6 *	18.2 ± 0.7 *	17.3 ± 1.1	57.3		<0.001
Year of practice	7.4 ± 2.0	8.5 ± 1.4	7.9 ± 13	7.9 ± 1.7	2.17		>0.05
Height (cm)	183.9 ± 7.7 ^¶^	187.8 ± 6.5	171.2 ± 6.4	181.0 ± 9.8	28.4		<0.001
Weight (Kg)	78.4 ± 9.5 ^¶^	85.7 ± 11.4	67.4 ± 7.2	77.2 ± 12.0	16.8		<0.001
BMI (Kg/m^2^)	23.1 ± 2.0	24.2 ± 2.8	23.0 ± 2.0	23.5 ± 2.3	1.7		0.187
TBW (l)	47.1 ± 4.0 ^†^	51.7 ± 4.9 ^†^	35.9 ± 2.7 ^†^	44.9 ± 7.7	73.8		<0.001
FFM (kg)	64.2 ± 5.5 ^†^	70.8 ± 6.4 ^†^	49.6 ± 3.2 ^†^	61.6 ±10.2	77.9		<0.001
FM (kg)	14.4 ± 5.1	14.9 ±6.0	17.8 ± 5.4	15.6 ± 5.6	2.2		0.125
FM (%)	17.6 ± 4.8	16.8 ± 5.1	25.9 ± 5.6	20.1 ± 6.6	16.8		<0.001
FFMI (Kg/m^2^)	19.0 ± 1.5 ^¶^	20.1 ± 1.5 ^¶^	16.9 ± 0.8	18.7 ± 1.8	25.7		<0.001
FMI (Kg/m^2^)	4.1 ± 1.3 ^¶^	4.2 ± 1.6 ^¶^	6.0 ± 1.7	4.8 ± 1.8	8.7		0.001
BCM (Kg)	38.8 ± 3.8 ^†^	43.8 ± 3.4 ^†^	29.1 ± 2.1 ^†^	37.3 ± 6.9		40.4	<0.001
R/H (Ω/m)	269.1 ± 25.5 ^†^	246.2 ± 18.8 ^†^	331.9 ± 28.8 ^†^	282.2 ± 43.6		35.7	<0.001
Xc/H (Ω/m)	35.4 ± 6.6 ^¶^	34.3 ± 7.4 ^¶^	41.5 ± 9.0	37.1 ± 8.1	11.7		<0.001
PA (°)	7.5 ± 0.5	7.9 ± 0.7 ^¶^	7.1 ± 0.7	7.5 ± 0.7	7.0		0.002
ECW: TBW	41.6 ± 3.8 ^‡^	38.3 ± 2.2	41.2 ± 2.8 ^‡^	40.4 ± 3.3		9.7	0.008

BMI: body mass index; TWB: total body water; FFM: fat-free mass; FM: fat mass; FFMI: fat-free mass index; FMI: fat mass index; BCM: body cellular mass; R/H: resistance/height; Xc/H: reactance/height; PA: phase angle; ECW:TBW: extracellular water/total body water ratio. * Significantly different from males U17; ^¶^ significantly different from females U19; ^†^ significant difference among the three groups; ^‡^ significantly different from males U19; BMC, R/H, and ECW: TBW were analyzed using the Kruskal–Wallis test due to non-normal distribution.

**Table 2:** Anthropometric, body composition, and bioelectrical data of the whole sample of young male and according to the positional roles.

Variable	Goalkeepers *n* = 6	Backs *n* = 9	Wingers *n* = 9	Pivots *n* = 7	Centers *n* = 6	Whole Sample *n* = 37	Χ **^2^**	*p*-Value
Age (years)	17.6 ± 1.4	17.5 ± 1.2	17.4 ± 1.3	17.3 ± 1.1	17.8 ± 1.3	17.5 ± 1.2	1.0	0.963
Height (cm)	188.8 ± 3.0	189.1 ± 6.3	179.8 ± 7.6 *^¶†^	190.6 ± 3.7 ^‡^	181.2 ± 7.0	185.8 ± 7.3	13.4	0.01
Weight (Kg)	89.6 ± 12.6	83.9 ± 7.1	70.4 ± 7.4 *^¶†^	89.1 ± 7.5 ^‡^	80.4 ± 8.9	82.0 ± 11.0	15.8	0.003
BMI (Kg/m^2^)	25.2 ± 3.8	23.5 ± 1.4	21.7 ± 1.1 ^¶†‡^	24.6 ± 2.6	24.5 ± 2.2	23.7 ± 2.5	9.7	0.046
TBW (l)	52.3 ± 7.4	50.2 ± 2.9	44.7 ± 3.7 *^¶†‡^	51.4 ± 3.8	49.7 ± 4.0	49.3 ± 5.0	11.0	0.027
FFM (kg)	71.2 ± 9.7	68.6 ± 4.3	61.4 ± 4.8 *^¶†^	70.1 ± 5.2	67.7 ± 6.2	67.4 ± 6.7	10.8	0.029
FM (kg)	18.4 ± 5.5	15.3 ± 4.1	8.9 ± 4.1 *^¶†^	19.0 ± 3.6 ^‡^	12.7 ± 3.5	14.5 ± 5.5	14.7	0.005
FM (%)	20.4 ± 4.5	18.9 ± 3.8	12.4 ± 4.4 *^¶†^	21.2 ± 2.8 ^‡^	15.6 ± 3.2	17.3 ± 4.9	15.5	0.004
FFMI (Kg/m^2^)	20.0 ± 2.7	19.2 ± 0.8	19.0 ± 0.8 ^‡^	19.3 ± 1.9	20.6 ± 1.6	19.5 ± 1.6	5.9	0.208
FMI (Kg/m^2^)	5.2 ± 1.7	4.3 ± 1.1	2.7 ± 1.1 *^¶†^	5.2 ± 1.0 ^‡^	3.8 ± 1.0	4.1 ± 1.5	14.5	0.006
BCM (Kg)	42.0 ± 6.2	42.3 ± 3.5	37.9 ± 3.3 ^¶†^	42.4 ± 4.5	42.7 ± 3.3	41.3 ± 4.4	7.7	0.102
R/H (Ω/m)	252.5 ± 43.2	257.0 ± 14.2	271.1 ± 21.3 ^‡^	257.4 ± 24.4	245.9 ± 18.9	258.0 ± 25.0	5.4	0.246
Xc/H (Ω/m)	31.5 ± 4.8	35.3 ± 2.7	37.4 ± 4.2 *	33.6 ± 2.3	35.6 ± 4.3	34.9 ± 4.0	7.2	=0.127
PA (°)	7.1 ± 0.4	7.8 ± 0.6 *	7.8 ± 0.7 *	7.5 ± 0.6 ^‡^	8.2 ± 0.4 *	7.7 ± 0.6	11.6	=0.018
ECW: TBW	42.0 ± 3.1	39.5 ± 3.6	39.5 ± 3.8	40.6 ± 3.0	39.0 ± 3.9	40.0 ± 3.5	5.9	=0.204

BMI: body mass index; TWB: total body water; FFM: fat-free mass; FM: fat mass; FFMI: fat-free mass index; FMI: fat mass index; BCM: body cellular mass; R/H: resistance/height; Xc/H: reactance/height; PA: phase angle; ECW:TBW: extracellular water/total body water ratio. * Significantly different from goalkeepers; ^¶^ significantly different from backs; ^†^ significantly different form pivots; ^‡^ significantly different form centers.

## Data Availability

Data are available on reasonable request from the authors.
